# Supplementary material for: PTHrP Modulates the Proliferation and Osteogenic Differentiation of Craniofacial Fibrous Dysplasia-Derived BMSCs
Source: Int J Mol Sci. 2023 Apr 20;24(8):7616. doi: 10.3390/ijms24087616 (PMC10146947; doi:10.3390/ijms24087616)
Supplement: Supplementary file 1 [file ijms-24-07616-s001.zip › ijms-2348694-supplementary.pdf]

Supplemental Table

Table S1. Antibodies Supplemental Table

Product name	Host species	Source and Catalog	Application and Dilution
Runx2	Rabbit	CST #12556	WB (1:1,000)
Runx2	Mouse	Abcam ab76956	IHC (1:200)
OCN	Rabbit	ABclonal A6205	IHC (1:500), WB (1:500)
Active β-Catenin	Rabbit	CST #19807	IHC (1:500), WB (1:1,000), IF (1:1,000)
PTHrP	Rabbit	ORIGENE TA334682	IHC (1:200), WB (1:500), IF (1:800)
TCF1/TCF7	Rabbit	CST #2203	WB (1:1,000)
p-CREB	Rabbit	CST #9198	WB (1:1,000)
CREB	Rabbit	CST #9197	WB (1:1,000), ChIP (1:50)
p-GSK-3β	Rabbit	CST #12456	WB (1:1,000)
GSK-3β	Rabbit	CST #5558	WB (1:1,000)
P-PKA C	Rabbit	CST#5561	WB (1:1,000)
PKA C	Rabbit	CST#5842	WB (1:1,000)
BrdU	Rabbit	ABclonal A20304	IF (1:400)
GAPDH	Mouse	ZSGB-BIO TA-08	WB (1:1,000)

Table S2. Biological Modulators Supplemental Table

**Modulator**	**Source, Catalog**	**Solvent**
PTHrP	Synthesized by Jiangsujitai, China<br>CAS:213779-11-4	PBS
6-TG	Selleck, S1774	DMSO
XAV-939	Selleck, S1180	DMSO
db-cAMP	Selleck, S7858	α-MEM
H89	Beyotime, S1643	DMSO
KG-501	Selleck, S8409	DMSO
IBMX	Selleck, S5836	DMSO
Wnt3a	Chemstan, CSP00225	PBS

Table S3. ChIP qPCR primer information

PTHLH ChIP P1	Forward	GGGCTCAAAATTTAAGAAAGGACCAT
	Reverse	ACAACCTCTCAACCTGAAGTCAATC
PTHLH ChIP P2	Forward	TGTTACTTGGGGTTTGAAGGC
	Reverse	AGACTTGAGAGGAGGCTGTTG
