# Supplementary material for: PTHrP Modulates the Proliferation and Osteogenic Differentiation of Craniofacial Fibrous Dysplasia-Derived BMSCs
Source: Int J Mol Sci. 2023 Apr 20;24(8):7616. doi: 10.3390/ijms24087616 (PMC10146947; doi:10.3390/ijms24087616)
Supplement: Supplementary file 1 [file ijms-24-07616-s001.zip › Supplementary figures.pdf]

Patients information

	Lesion site	Gender	Age	Imaging	Amplicon sequencing
FD Patient1	Maxilla	Male	21	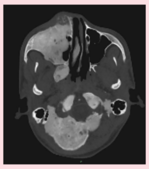	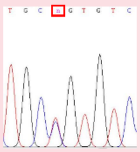 C→T
FD Patient2	Maxilla	Femal	22	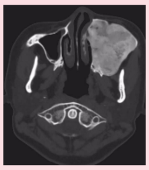	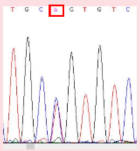 C→T
FD Patient3	Mandible	Male	18	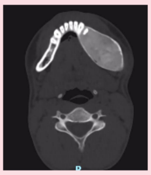	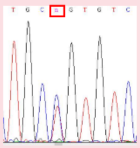 C→T
	**Specimen site**	**Gender**	**Age**	**Deformity type**	**Amplicon sequencing**
NC Patient1	Maxilla	Male	25	maxillary protrusion	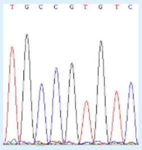
NC Patient2	Mandible	Male	20	mandibular protrusion	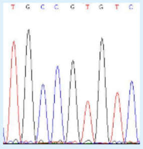
NC Patient3	Maxilla	Femal	23	maxillary protrusion	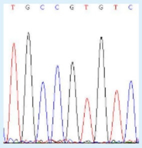

Figure S1. Patients clinical information and mutation analysis. Amplicon sequencing was completed by Ruibiotech, Beijing.

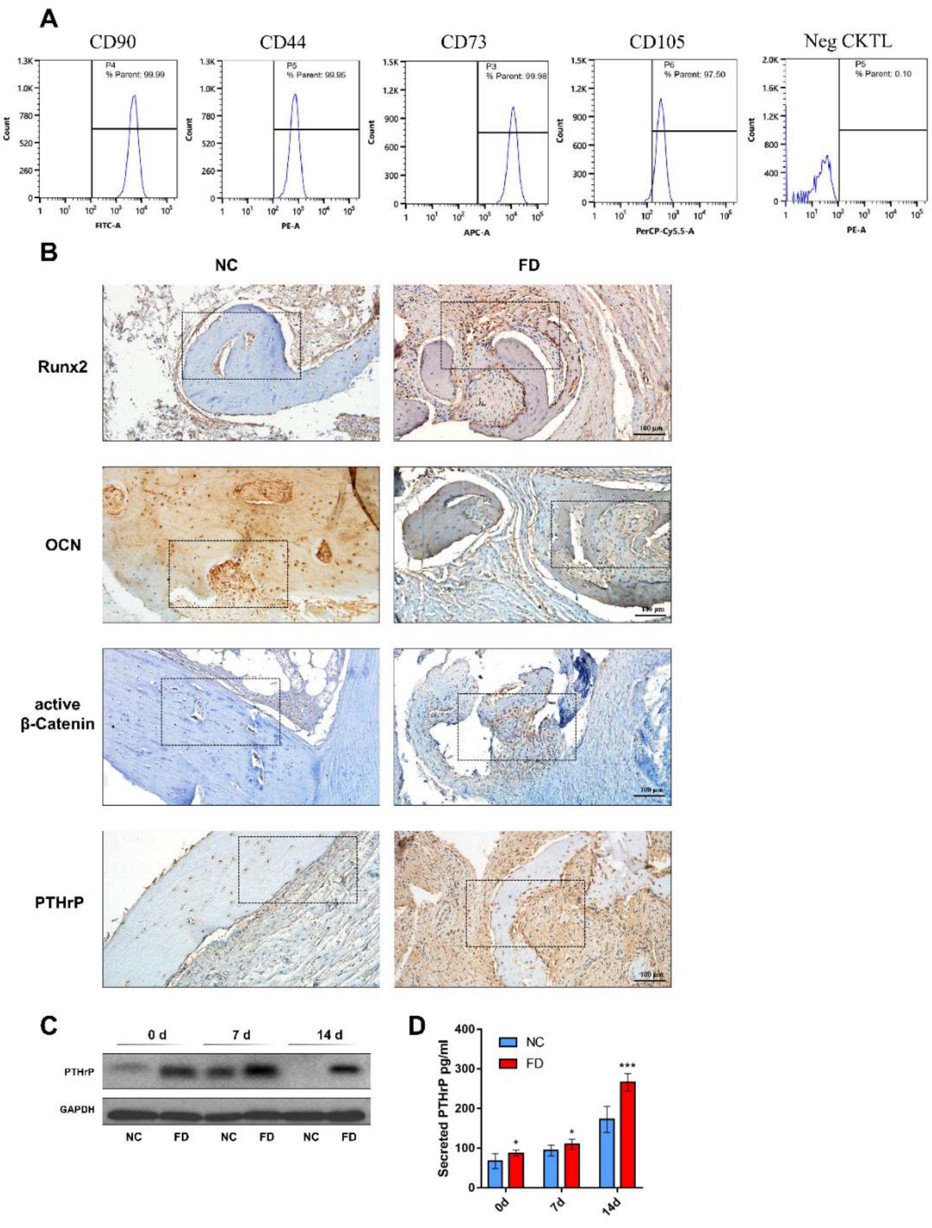

Figure S2. (A): Multipotent mesenchymal stromal cells identification was performed using BD StemflowTM 562245. Results showed that primary cells were positive for CD9, CD44, CD73, CD10, and negative for the Negative Cocktail (CD34, CD11b, CD19, CD45, HLA-DR). (B): 20× light microscope images of the IHC images in Figure 1A. (C): WB results of the changing trend of PTHrP during BMSCs

differentiation. (D): ELISA results of the changing trend of secreted PTHrP during BMSCs differentiation. Bars indicate means ± SD. $p > 0.05$, ; $p < 0.05$, *; $p < 0.001$, ***.

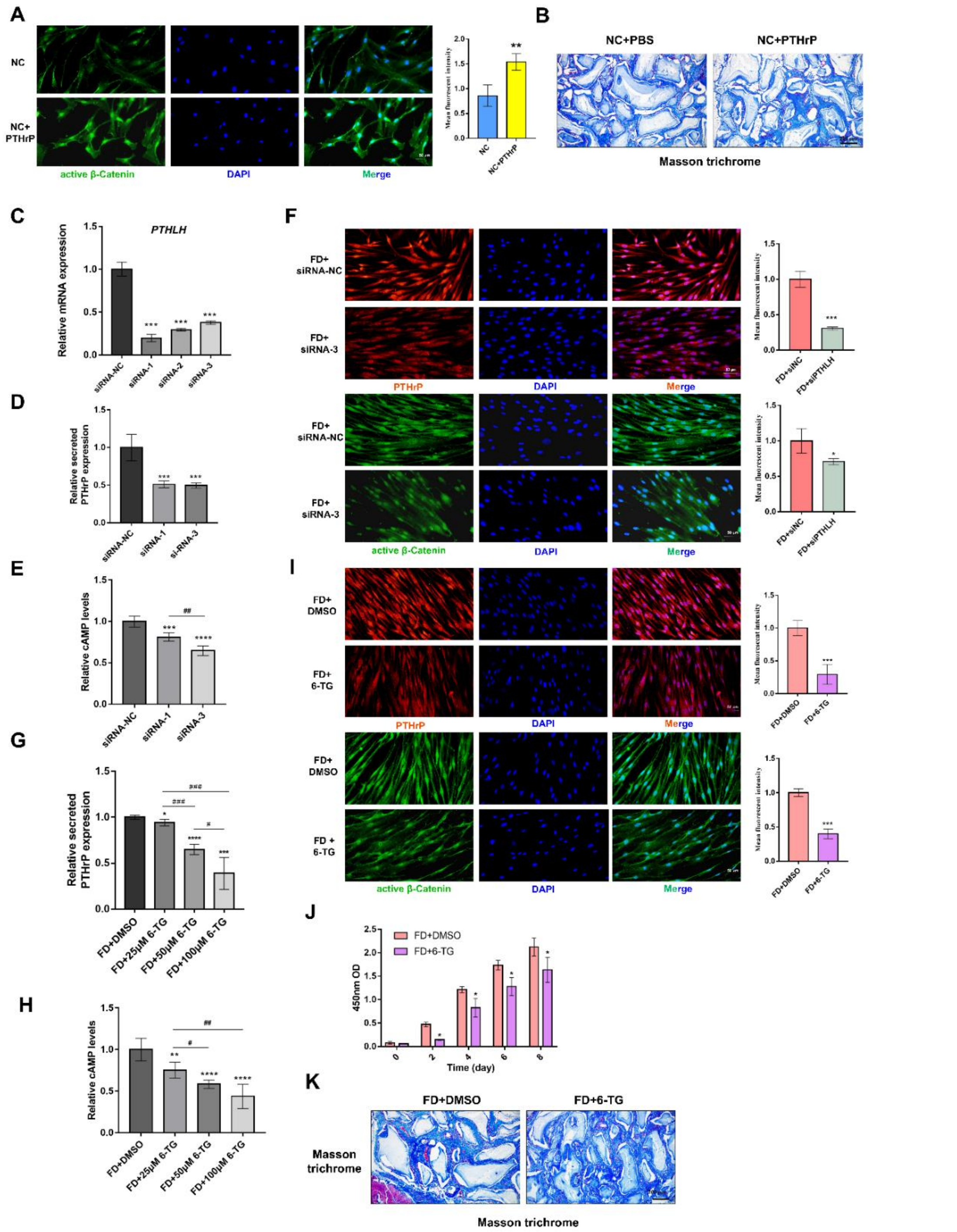

Figure S3. (A): Immunofluorescence staining changes of active β-Catenin in PTHrP

(200 nM) or PBS-treated NC BMSCs. (B): Masson trichrome staining of *in vivo* results. (C): The interference efficiency of three siRNA sequences was ascertained via qPCR. (D) ELISA results of relative secreted PTHrP in siRNA-treated FD BMSCs. (E): Relative cAMP levels in cells post-transfection with two independent siRNA. (F): Immunofluorescence staining changes of PTHrP and active β-Catenin in FD BMSCs when treated with si-*PTHLH*-RNA3. (G): ELISA results of secreted PTHrP with varying concentration of 6-TG treated FD BMSCs. (H): Relative cAMP levels in FD cells following treatment with concentrations of 6-TG ranging from 0 to 100 μM. (I): Immunofluorescence staining changes of PTHrP and active β-Catenin in FD BMSCs when treated with 6-TG (50 μM). (J): Proliferation capacity of FD BMSCs with or without 6-TG (50 μM) based on CCK8 assay. (K): Masson trichrome staining of *in vivo* results. Bars indicate means ± SD. $p > 0.05$, ; $p < 0.05$, */#; $p < 0.01$, **/##; $p < 0.001$, ***/###; $p < 0.0001$, ****

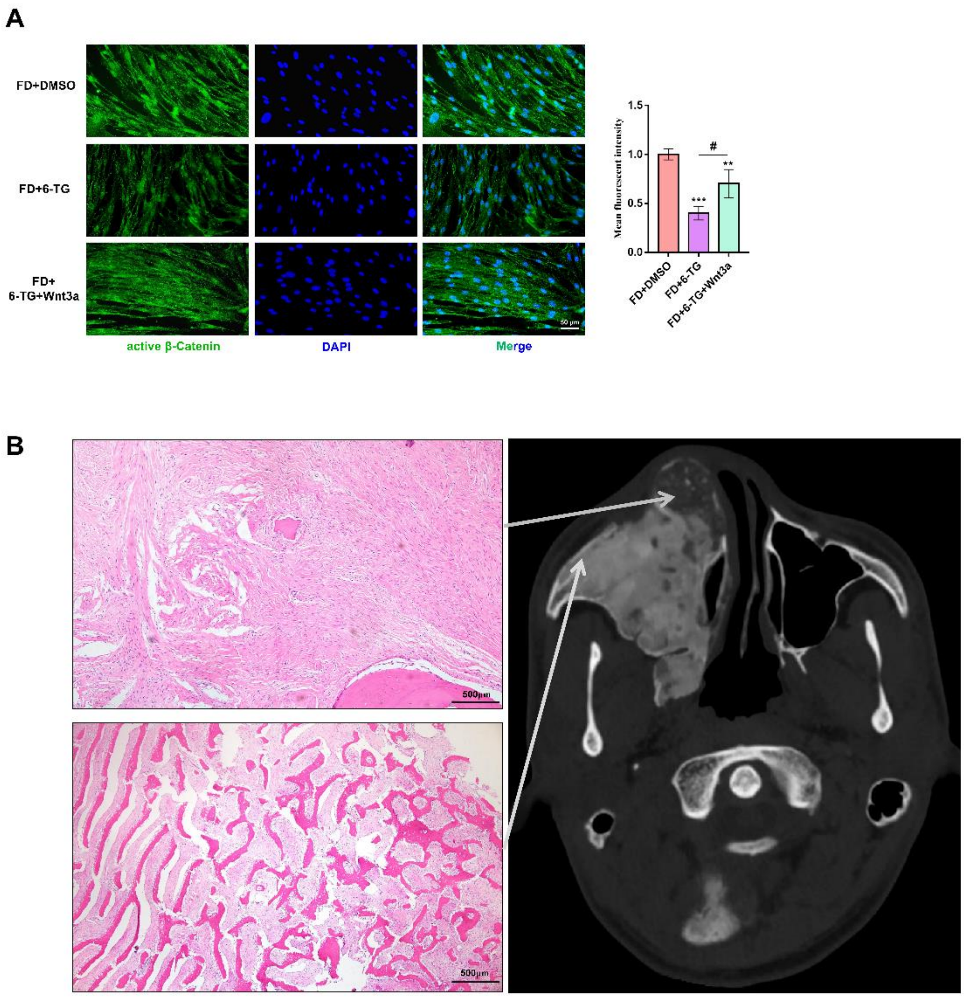

Figure S4. (A): Immunofluorescence staining changes of active β-Catenin in Wnt3a (100 ng/mL) and/or 6-TG (50 μM) treated FD BMSCs. (B):In the same patient, different CT gray values corresponded to distinct histological findings: Regions with

lower gray scale values exhibited dense fibrous tissue and sparse trabeculaes; areas with ground-glass appearance demonstrated characteristic FD histological features. (CT scanner: Optima CT520; GE Healthcare, Waukesha,WI) Bars indicate means ± SD. $p > 0.05$, ; $p < 0.05$, #; $p < 0.01$, **; $p < 0.001$, ***.
